# Supplementary material for: Detection of functional protein domains by unbiased genome-wide forward genetic screening
Source: Sci Rep. 2018 Apr 18;8:6161. doi: 10.1038/s41598-018-24400-4 (PMC5906580; doi:10.1038/s41598-018-24400-4)
Supplement: Supplementary file 1 — Supplementary Figures and Legends [file 41598_2018_24400_MOESM1_ESM.pdf]

Detection of functional protein domains by unbiased genome-wide forward genetic screening

Mareike Herzog[1,#], Fabio Puddu[1,#], Julia Coates[1], Nicola Geisler[1], Josep V Forment[1,†,*] and Stephen P. Jackson[1,*]

1 The Wellcome/CRUK Gurdon Institute and Department of Biochemistry, University of Cambridge, Tennis Court Road, CB2 1QN, Cambridge, UK

# these authors contributed equally to this work

* to whom correspondence should be addressed: Stephen P. Jackson, Tel: +44 1223 334088 (email: s.jackson@gurdon.cam.ac.uk); correspondence may also be addressed to: Josep V. Forment, Tel: +44 1223 334088 (email: j.forment@gurdon.cam.ac.uk)

† Current address: AstraZeneca, Oncology DNA damage response group, Hodgkin Building, 310 Cambridge Science Park Milton Road, CB4 0WG, Cambridge, UK (josep.forment@astrazeneca.com)

Supplementary Figures

Supplementary Figure 1. (a) Examples of two camptothecin resistant yeast strains, which each carry a large deletion in the *TOP1* gene. (b) Nonsense mutations are depicted as in **Figure 2D**. Superimposed is the frequency of codons that can be mutated to a stop codon by one nucleotide change. (c) Frameshift mutations are depicted as in **Figure 2E**. Above the locations of homopolymers of a length of at least 3nt in the *TOP1* gene are plotted, their length indicated on the y-axis.

Supplementary Figure 2. (a) Integrative Genomics Viewer (IGV) panels of the sequencing data for clones A9 and H10 showing the *Parp1* mutation (*Parp1 Δ341*) that did not pass the filters. (b) Integrative Genomics Viewer (IGV) panels of the sequencing data for clones A7 and B7 showing the *Parp1* mutation (*Parp1 R138C*) that did not pass the filters.

Supplementary Figure 3. (a) Western blots for PARP1 protein for olaparib resistant clones. (b) PARP1-DNA binding assays in olaparib resistant clones. Two exposure each are shown.

Figure S1

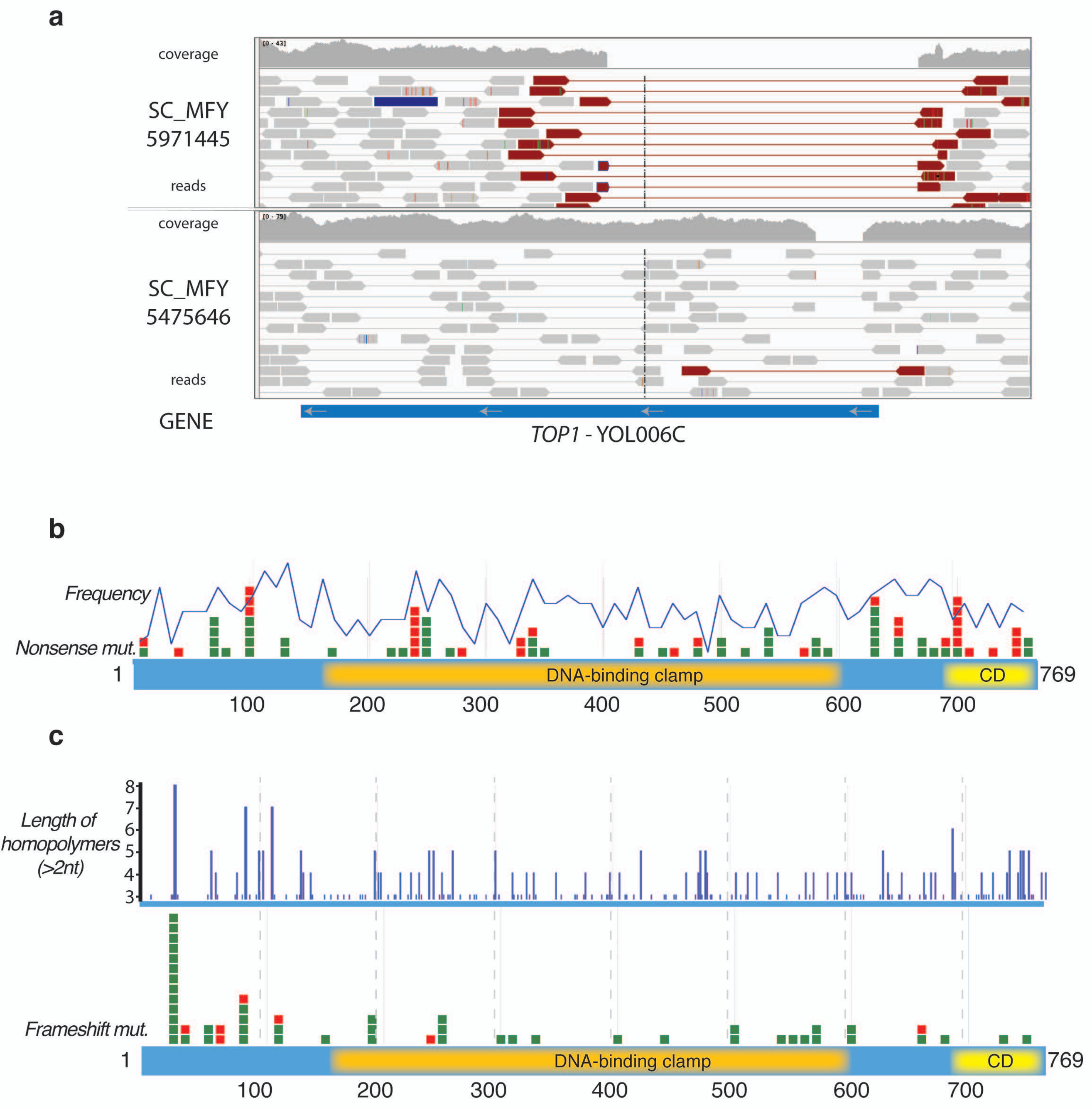
a
coverage
[0 - 43]
SC_MFY
5971445
reads
coverage
[0 - 79]
SC_MFY
5475646
reads
GENE
TOP1 - YOL006C
b
Frequency
Nonsense mut.
1
DNA-binding clamp
CD
769
100
200
300
400
500
600
700
c
Length of
homopolymers
(>2nt)
8
7
6
5
4
3
Frameshift mut.
1
DNA-binding clamp
CD
769
100
200
300
400
500
600
700

Figure S2

a

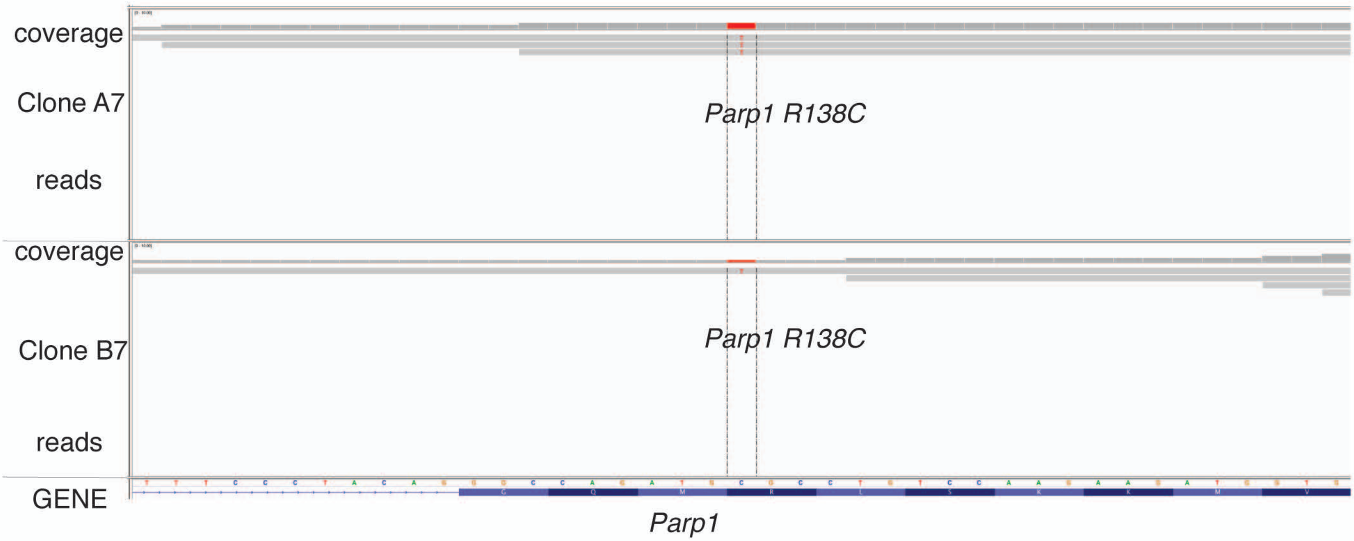
coverage
Clone A7
reads
Parp1 R138C
coverage
Clone B7
reads
Parp1 R138C
GENE
Parp1

b

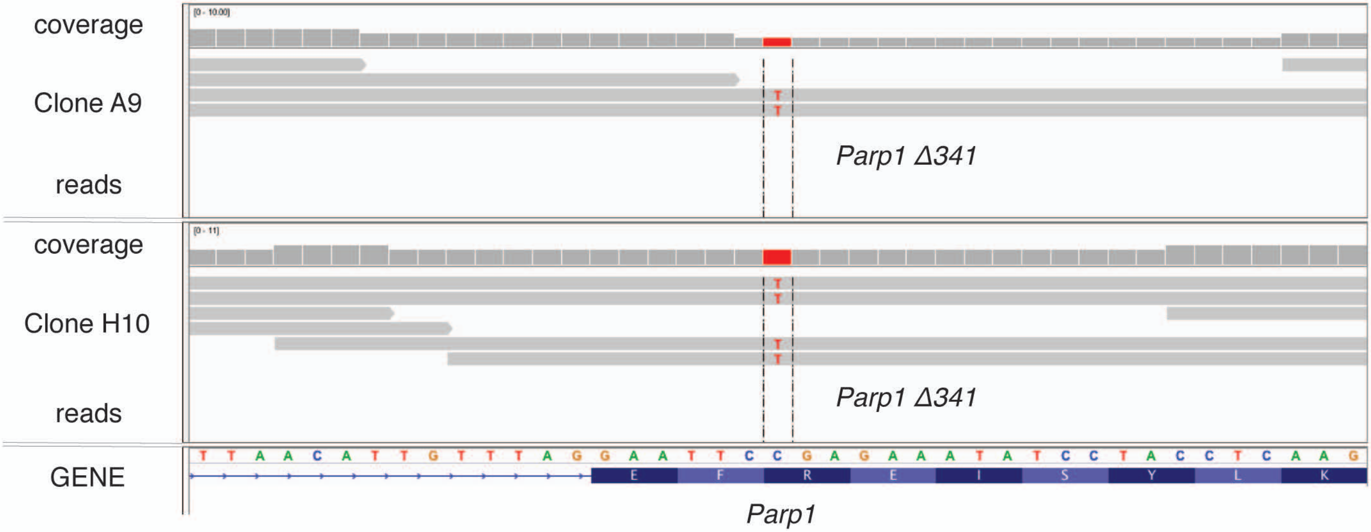
[0 - 10.00]
coverage
Clone A9
reads
Parp1 Δ341
[0 - 11]
coverage
Clone H10
reads
Parp1 Δ341
GENE
T T A A C A T T G T T T A G G A A T T C C G A G A A A T A T C C T A C C T C A A G
E F R E I S Y L K
Parp1

Figure S3

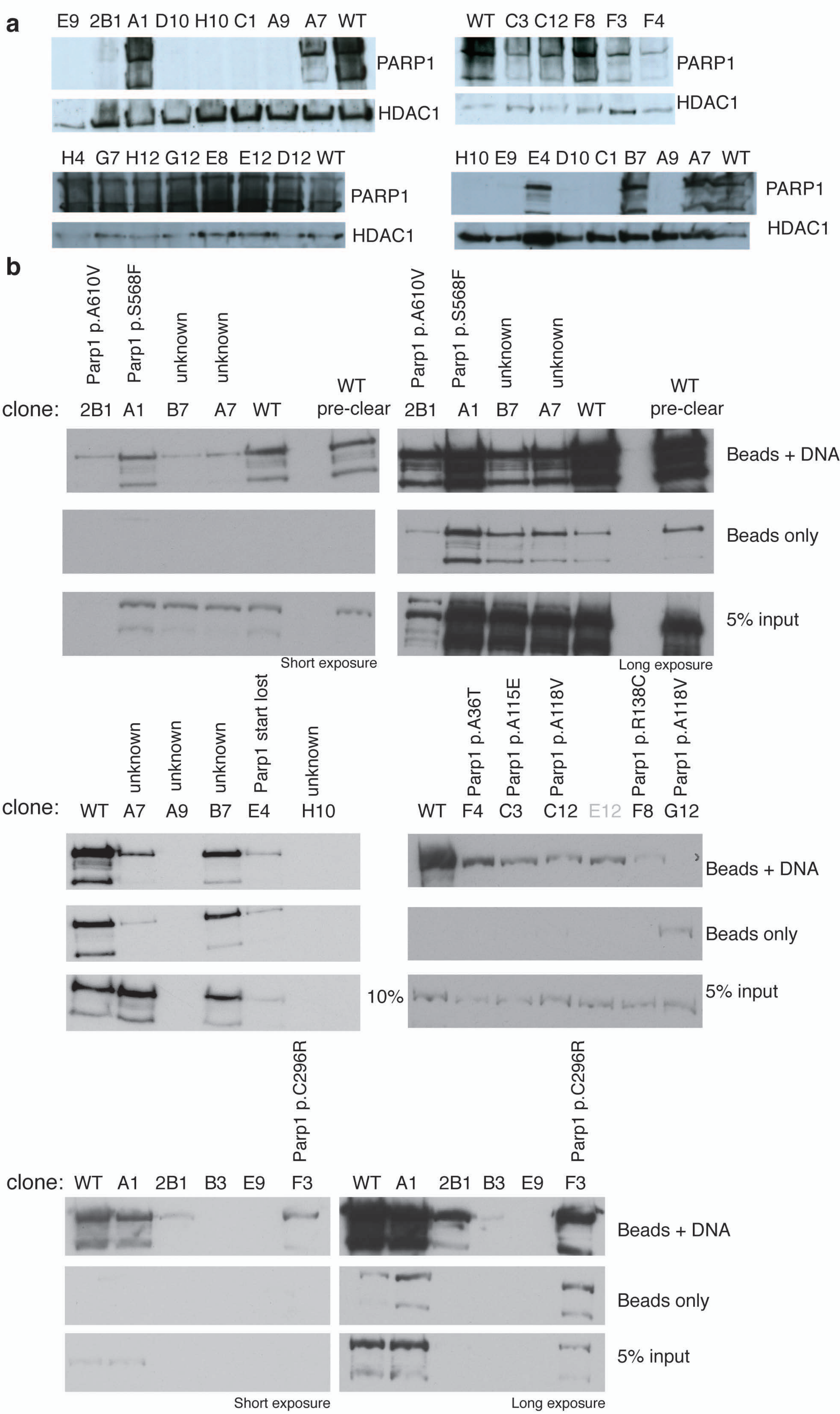
a
E9 2B1 A1 D10 H10 C1 A9 A7 WT
PARP1
HDAC1
WT C3 C12 F8 F3 F4
PARP1
HDAC1
H4 G7 H12 G12 E8 E12 D12 WT
PARP1
HDAC1
H10 E9 E4 D10 C1 B7 A9 A7 WT
PARP1
HDAC1
b
Parp1 p.A610V
Parp1 p.S568F
unknown
unknown
clone: 2B1 A1 B7 A7 WT
WT pre-clear
Beads + DNA
Beads only
5% input
Short exposure
Long exposure
unknown
unknown
unknown
Parp1 start lost
unknown
clone: WT A7 A9 B7 E4 H10
10%
Parp1 p.A36T
Parp1 p.A115E
Parp1 p.A118V
Parp1 p.R138C
Parp1 p.A118V
WT F4 C3 C12 E12 F8 G12
Parp1 p.C296R
clone: WT A1 2B1 B3 E9 F3
